# Factors associated with response to patient-reported outcome measures: a systematic review of systematic and scoping reviews, and meta-analyses

**DOI:** 10.1007/s11136-026-04314-9

**Published:** 2026-06-22

**Authors:** Sebastiaan Tjènne Peters, Joene Rutten, Yara Eline van Kooij, Ruud Willem Selles, Harm Pieter Slijper, Hand-Wrist Study Group, Robbert Maarten Wouters

**Affiliations:** 1 Department of Plastic, Reconstructive and Hand Surgery, Erasmus MC, University Medical Center Rotterdam, Erasmus MC Rotterdam, Dr. Molewaterplein 40, 3015 GD Rotterdam, The Netherlands; 2 Department of Rehabilitation Medicine, Erasmus MC, University Medical Center Rotterdam, Rotterdam, The Netherlands; 3 Hand and Wrist Center, Xpert Clinics, Rotterdam, The Netherlands; 4 Center for Hand Therapy, Xpert Handtherapie, Utrecht, The Netherlands

**Keywords:** Compliance, Digital, Factors, PROMs, Response

## Abstract

**Purpose:**

This study aimed to identify and synthesize factors associated with response to digitally collected patient-reported outcome measures (PROMs) among adult patients, to inform efforts to improve response rates.

**Methods:**

We performed a systematic review of systematic and scoping reviews across five databases. Risk of bias was assessed using A MeaSurement Tool To Assess Systematic Reviews (AMSTAR) and included in a best-evidence synthesis. Additionally, a meta-analysis was conducted using the primary studies from the included reviews that specifically reported on digitally collected PROMs.

**Results:**

We identified 43 factors positively, negatively, or not associated with response to digital PROMs, clustered in six domains. Some were modifiable, including the overload or overlap of questions, PROMs irrelevant to the patient, automatic reminders, system usability, and clinicians’ follow-up with PROMs. Others were non-modifiable, such as race, language barriers, visual impairments, and comorbidities; many of these factors are shaped by patients’ broader social and health-related circumstances and may therefore point to underlying structural or contextual barriers (e.g., limited access to resources, lower literacy, or health-related limitations). Such barriers may ultimately contribute to unequal access to care and are potentially actionable.

**Conclusions:**

Factors associated with response to digitally collected PROMs can be clustered into six domains: sociodemographic characteristics, physical health, psychosocial status, PROM characteristics, technological factors, treatment characteristics, and external influences. Although not all factors are directly modifiable, they can inform targeted and tailored interventions to improve response rates across diverse populations and support more equitable access to care. This review provides a structured overview to guide such efforts. Potential interventions could include language simplification, item reduction, follow-up during consultation, tailored reminders, and interface adaptations for patients with specific needs.

**Supplementary Information:**

The online version contains supplementary material available at https://doi.org/10.1007/s11136-026-04314-9.

## Abstract (--)

1. Digital questionnaires (called patient-reported outcome measures) ask patients how they feel and how they function in daily life. This helps clinicians understand patients better and improve care. However, many patients do not fill in these questionnaires, especially when these questionnaires are digital. This makes it harder to use the information in daily care. To make it easier for patients to fill in these questionnaires, we need to understand why some patients fill them in while others do not, but this is not always clear.
2. We wanted to find out why some patients complete these digital questionnaires and others do not.
3. We looked at results from earlier studies and combined their findings to get a clear overview of what is already known.
4. We found that many factors can affect whether patients complete questionnaires. Some of these can be changed, such as making systems easier to use or reducing the number of questions. Others cannot be changed, such as language difficulties, health problems, or access to technology.
  This means that some patients may find it harder to take part. To improve response rates, we need to design systems that are easier to use and better suited to all patients. This means we can take action, for example, by using simpler language and giving support that fits what patients need.

## Introduction

Over the past fifteen years, routine outcome measurement, including digitally collected patient-reported outcome measures (PROMs), has become increasingly central to value-based healthcare [1, 2]. By integrating the patient’s voice through PROMs, clinicians can capture subjective experiences and personal insights that are often overlooked by traditional clinical measures [3–5]. This can improve healthcare quality by facilitating shared decision-making, predicting outcomes, and monitoring treatment progression over time [4–8]. Ultimately, routinely collected PROMs can lead to improved patient experiences, more positive outcome expectations, and healthcare better tailored to patients’ needs [1, 5, 8–11].

Digitally collected PROMs facilitate more efficient data collection by enabling questionnaires to be completed remotely, outside the clinical setting. This enables access to outcomes prior to consultations and supports longitudinal monitoring, potentially reducing unnecessary follow-up visits [12–16]. Despite the potential benefits of using digitally collected PROMs, achieving high response rates remains challenging [17–20]. When response rates are low, clinicians cannot rely on PROM outcomes being available, which limits the practical implementation of value-based healthcare. Consequently, the effort and resources invested in collecting PROMs may not meaningfully contribute to improving daily clinical care. In other words, high response levels are needed [21].

To achieve these high response rates, optimization often requires targeted interventions and strategies informed by an understanding of the factors that influence patient response on digitally collected PROMs [22, 23]. Multiple studies—often summarized in systematic and scoping reviews of specific patient populations—have explored these factors [24–26]. However, many of these reviews are based on small sample sizes or limited studies, which undermines the generalizability of their findings [24–26]. In addition, inconsistent findings suggest that the factors influencing response rates might be context-specific, thereby complicating the development of effective, broadly applicable strategies to improve response rates [24–26].

A synthesis of multiple systematic and scoping reviews can provide a broader perspective on the factors associated with patient response, highlighting those with large effect sizes, as well as identifying consistent and conflicting or context-specific findings in the literature. This approach may offer a more generalizable and comprehensive understanding, alongside a stronger body of evidence regarding the factors associated with response to digitally collected PROMs [27]. Ultimately, this can inform the development of targeted interventions and strategies to improve response. Therefore, this systematic review and meta-analysis of existing systematic and scoping reviews aimed to identify factors associated with response to digitally collected PROMs in adult patients from any population.

## Methods

### Design

We conducted a systematic review of systematic and scoping reviews [27] and performed a meta-analysis.

following the Preferred Reporting Items for Systematic Reviews and Meta-Analyses (PRISMA) guidelines for systematic reviews and meta-analyses [28].

### Information sources and search strategy

We performed an electronic search across five databases (MEDLINE, Embase, Web of Science, Cochrane Central Register of Controlled Trials, and Epistemonikos) from inception until April 18, 2024. The search strategy was developed and refined in collaboration with an experienced medical librarian. To ensure sensitivity, we performed an initial validation by verifying whether a set of known relevant studies was retrieved. The search was subsequently updated to include studies published up to May 27, 2025. Additionally, we reviewed the top 200 results from Google Scholar. We did this because, unlike other databases, Google Scholar searches the full text of articles, potentially identifying relevant studies that may not appear in database searches [29]. The search strategy included the keywords “patient-reported outcome measure,” “response,” “digital,” and “review,” along with their relevant variants and synonyms, to identify systematic and scoping reviews, with or without meta-analyses, that report factors associated with response to digitally collected PROMs. The complete search strategy is presented in Appendix 1.

### Study selection

After removing duplicates, two reviewers (SP and JR) independently screened articles for eligibility based on their titles and abstracts using Covidence, systematic review management software [30]. If an article appeared potentially eligible, SP and JR reviewed the full text for eligibility. Following both selection rounds, a consensus meeting was held to resolve any remaining differences.

### Eligibility criteria

Studies were eligible for inclusion if they met the following criteria: (1) the study design was a systematic or scoping review with or without meta-analyses; (2) assessed factors that are associated with response to digitally distributed PROMs; and (3) concerned adult patients with any medical condition. We excluded studies if they did not meet these criteria, or if (1) the text was non-English, (2) the study was not peer-reviewed (e.g., conference abstract), or (3) the full text was unavailable. We included both systematic and scoping reviews, as they address the same research question and both employ systematic search strategies to identify relevant studies. Excluding them would leave out important evidence. We weighted the methodological rigor of these studies in the risk of bias assessment.

### Risk of bias assessment

Two reviewers (SP and JR) independently assessed the risk of bias in the included reviews using the *A MeaSurement Tool To Assess Systematic Reviews* (AMSTAR) [31]. The AMSTAR assesses the risk of bias using eleven items. Reviews scoring eight or higher were categorized as having a low risk of bias, whereas scores ranging from five to seven indicated a moderate risk of bias. Scores of four or lower were considered high risk of bias [31]. We chose the original version of the AMSTAR tool over AMSTAR-2 because it allows calculation of an overall methodological quality score, enabling classification of reviews for our best-evidence synthesis [32]. Additionally, it demonstrates favorable psychometric properties, including inter-rater reliability and construct validity [33]. Although AMSTAR-2 provides a more detailed domain-based assessment, it does not generate an overall score [32]. Discrepancies between reviewers were discussed during a consensus meeting, and if agreement could not be reached, a third reviewer (RW) was consulted.

### Data extraction

Data were extracted by SP and JR using a predefined data extraction form. The following data were extracted from the included reviews: (1) author; (2) year of publication; (3) study population; (4) number of studies included; (5) total number of participants in the included studies; (6) start and end date of the search strategy; (7) whether PROMs in the included studies of the reviews were collected fully or partially digitally; (8) aim of the study; and (9) factors associated with response. Additionally, if available, we manually extracted the corresponding odds ratios (ORs), confidence intervals (CIs), *p*-values, standard errors, and sample sizes from the primary studies that focused on digital PROM collection and were included in the reviews to perform a meta-analysis.

### Synthesis of results, meta-analysis, and data triangulation

We synthesized the findings of the included studies using two independent methods. First, we performed a best-evidence synthesis based on the review outcomes, including only those reviews that focused solely on digital PROM collection. We adapted the best-evidence synthesis guidelines from the Cochrane Collaboration Back Review Group’s systematic review criteria [34]. Specifically, we applied these criteria across the included reviews and determined the levels of evidence on the consistency of findings, methodological quality of the reviews, and the number of reviews reporting each factor. We categorized the results into one of four levels of evidence: strong, moderate, weak, or inconsistent; the predefined criteria are outlined in Table 1. If a factor was covered in only one review, and that review had already conducted a best-evidence synthesis, we adopted the level of evidence reported in that review.

**Table 1:** Best-evidence synthesis for a systematic review of systematic and scoping reviews

Level of evidence	Criteria
Strong evidence:	If level of evidence is determined*: strong evidence in at least one high-quality review *In absence of a level of evidence:* consistent findings in multiple (≥ 2) high- or moderate-quality reviews *or* consistent findings in one moderate and multiple (≥ 2) low-quality reviews
Moderate evidence:	If level of evidence is determined*: moderate evidence in one high- or moderate-quality review *In absence of a level of evidence:* consistent findings in one moderate-quality review **or** consistent findings in multiple (≥ 2) low-quality reviews
Weak evidence:	If level of evidence is determined*: Weak** evidence in one high- or moderate-quality review *In absence of a level of evidence:* consistent finding in one low-quality review
Inconsistent evidence:	Inconsistent findings between multiple (≥ 2) review (e.g., less than 75% of the studies presented results in the same direction) *or* inconsistent findings within one or more review

*Level of evidence is determined within the included study using a best-evidence synthesis; ** Some studies also use the terminology inconclusive, which is defined as weak in this best-evidence synthesis

Second, to complement the best-evidence synthesis, we conducted a meta-analysis of factors associated with response to digitally collected PROMs. This analysis was based on primary studies included in the reviews that (1) specifically reported on digitally collected PROMs and (2) reported odds ratios. Effect estimates were extracted directly from the primary studies rather than from the reviews. A factor had to be reported in multiple primary studies to be included in the meta-analysis, as pooling is not possible when a factor is reported in a single primary study. Factors reported only once were therefore excluded from the meta-analysis but were retained in the best-evidence synthesis and reported in the results. If the same primary study was identified in multiple systematic reviews, it was included only once in the meta-analysis. Additionally, corresponding confidence intervals, *p*-values, and/or standard errors had to be reported alongside the odds ratio to enable inclusion in the meta-analysis. We assessed heterogeneity using I^2^ [35]. We considered a fixed-effects model when heterogeneity was low (I^2^ < 30%) and a random-effects model when heterogeneity was high [36]. We created funnel plots for the included factors to assess publication bias [37].

We combined the best-evidence synthesis and meta-analysis results using a triangulation protocol [38]. The first step involved organizing the data at the factor level. Second, we performed convergent coding, which included separately comparing the results of the best-evidence synthesis and meta-analysis. Finally, we conducted a comprehensive comparison, combining the results of both methods. The final interpretation of the strength and direction of effects was performed independently by two reviewers (SP and JR). In this step, the results of the best-evidence synthesis (as defined in Table 1) and the meta-analysis were compared, taking into account consistency between methods, methodological quality of the included reviews (based on AMSTAR), and the statistical significance and direction of pooled estimates, as well as the precision of the estimates as reflected by the width of the 95% confidence intervals. Discrepancies were resolved through discussion during meetings with the research team until consensus was reached.

In addition, identified factors were grouped into broader conceptual domains to facilitate interpretation. This clustering was performed through an iterative, team-based process during the stages of best-evidence synthesis and triangulation. This clustering was performed through an iterative, team-based process, with consensus reached during multiple research team meetings and supported by expert input.

## Results

### Study selection, study characteristics, and risk of bias assessment

**Fig. 1:**
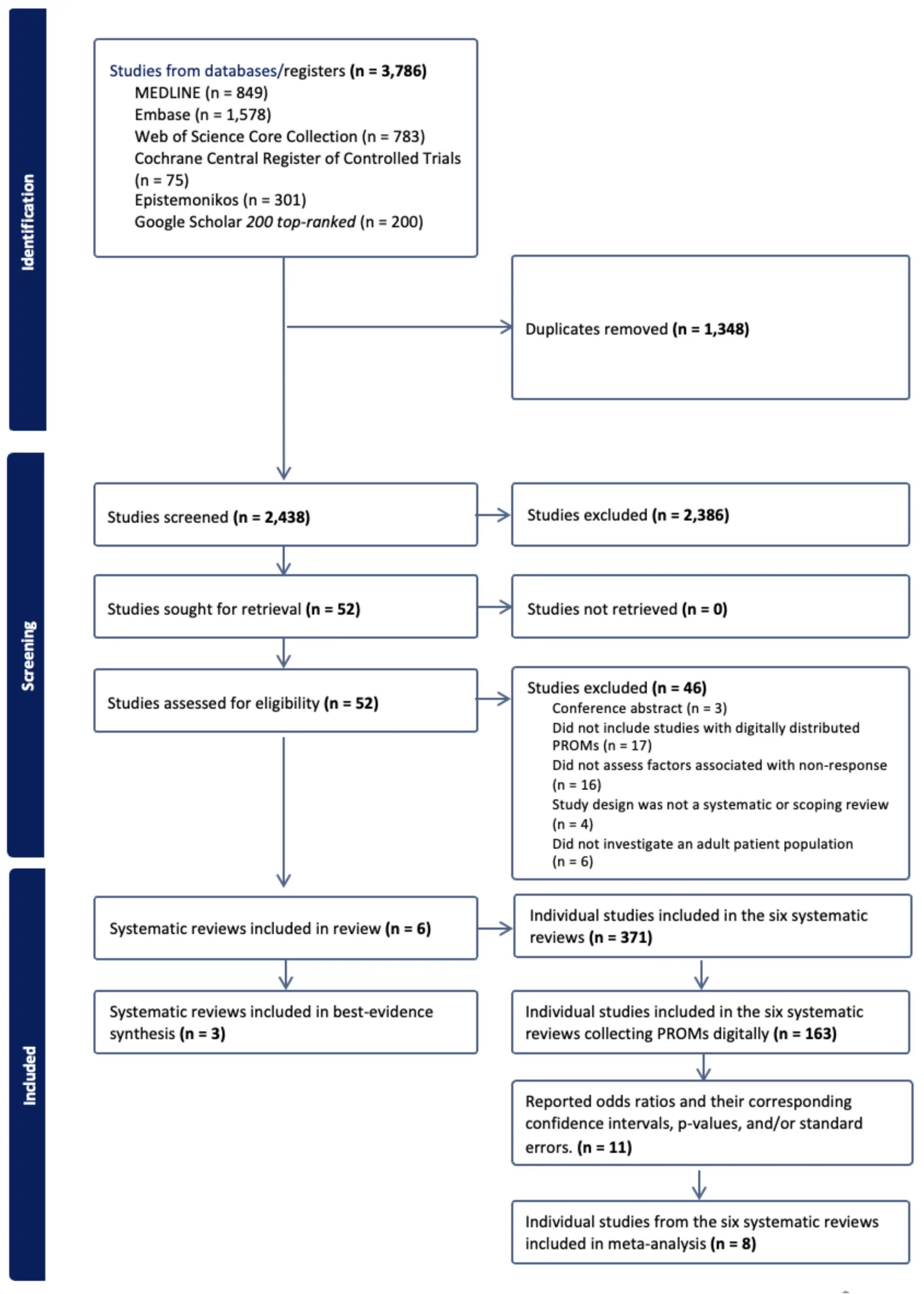
Preferred Reporting Items for Systematic Reviews and Meta-Analyses (PRISMA) flowchart illustrating the search and study selection process

The initial search in MEDLINE, Embase, Web of Science, Cochrane Central Register of Controlled Trials, Epistemonikos, and Google Scholar identified 3,014 potential studies. After removing duplicates, 2,438 studies were eligible for screening based on their titles and abstracts, resulting in 52 studies that qualified for full-text assessment. Following this assessment, we included six reviews [19, 24–26, 39, 40]. A detailed description of the characteristics of the included reviews is provided in Table 2*.* The PRISMA flowchart illustrating the search and selection process is presented in Fig. 1. Studies excluded at the full-text stage are listed in Appendix 2, along with the reasons for exclusion.

**Table 2:** Study characteristics of the included reviews

Author (year)	Risk of bias	Study population	Included studies (n)	Total number of participants (n)	Start and end date search strategy	Included studies collected PROMs digitally (yes/partially)	Aim
Cho et al. [26]	Moderate	Cancer	33	7,382	January 2010–March 2020	Yes	To understand the acceptance and use of home-based Electronic Symptom Self-Reporting by patients with cancer and identify associated facilitators and barriers
Levens et al. [25]	Moderate	Orthopaedic surgery	97	Unclear	Inception–October 2022	Partially*	To investigate orthopaedic patient compliance with patient-reported outcome measures and identify factors that improve response rates
Nielsen et al. [39]	Moderate	Long-term conditions in outpatient care	51	14,712	January 2008–January 2019	Yes	To review empirical studies of the use of digitally administered patient-reported outcome in routine care and examine the stated reasons for patients’ non-use of digital patient-reported outcome
Ruseckaite et al. [19]	Moderate	Conditions with clinical registries	141	Unclear	Inception–August 2022	Partially	To describe response rates (RR) to PROMs in clinical registries and databases and to examine the trends over time, and how they change with the registry type, region and disease or condition captured
Van Egdom et al. [40]	Moderate	Breast cancer	34	14,901	Inception–November 2017	Partially	To give an overview of patient-reported outcome measures administration methods and their facilitators and barriers in breast cancer clinical practice
Wiegel et al. [24]	Low	Chronic diseases	5	757	Inception–June 2020	Yes	To identify factors quantitatively associated with adherence to telemonitoring by repetitive electronic patient-reported outcome measures in patients with chronic diseases in all studies

Five studies had a moderate risk of bias and did not perform a best-evidence synthesis to formulate conclusions based on the risk of bias [19, 25, 26, 39, 40]. Only the study by Wiegel et al. had a low risk of bias and incorporated a risk of bias assessment into their synthesis (Table 2) [24]. None of the included reviews conducted a meta-analysis. The full risk of bias assessment, including item-level AMSTAR scores for each included review, is detailed in Appendix 3.

Three reviews solely focused on digital PROM collection and were included in the best-evidence synthesis [24, 26, 39], whereas the other three also assessed non-digital PROM collection and were not included in the best-evidence synthesis. Furthermore, out of the 371 primary studies included in the reviews, eight identified factors with odds ratios were reported in multiple studies and were included in the meta-analysis [41–48].

### Best evidence synthesis

Forty-two factors were identified as either associated or not associated with response [24, 26, 39]. These factors were operationalized based on their definitions in the included studies; detailed definitions are provided in Appendix 4. We clustered these factors into six domains: (1) sociodemographic characteristics, (2) physical health and psychosocial status, (3) PROM characteristics, (4) technological factors, (5) treatment characteristics, and (6) external influences. A complete overview of the identified factors, their associations with response as reported in the individual reviews, and the combined strength of the evidence based on the best-evidence synthesis across reviews is presented in Table 3.

**Table 3:**
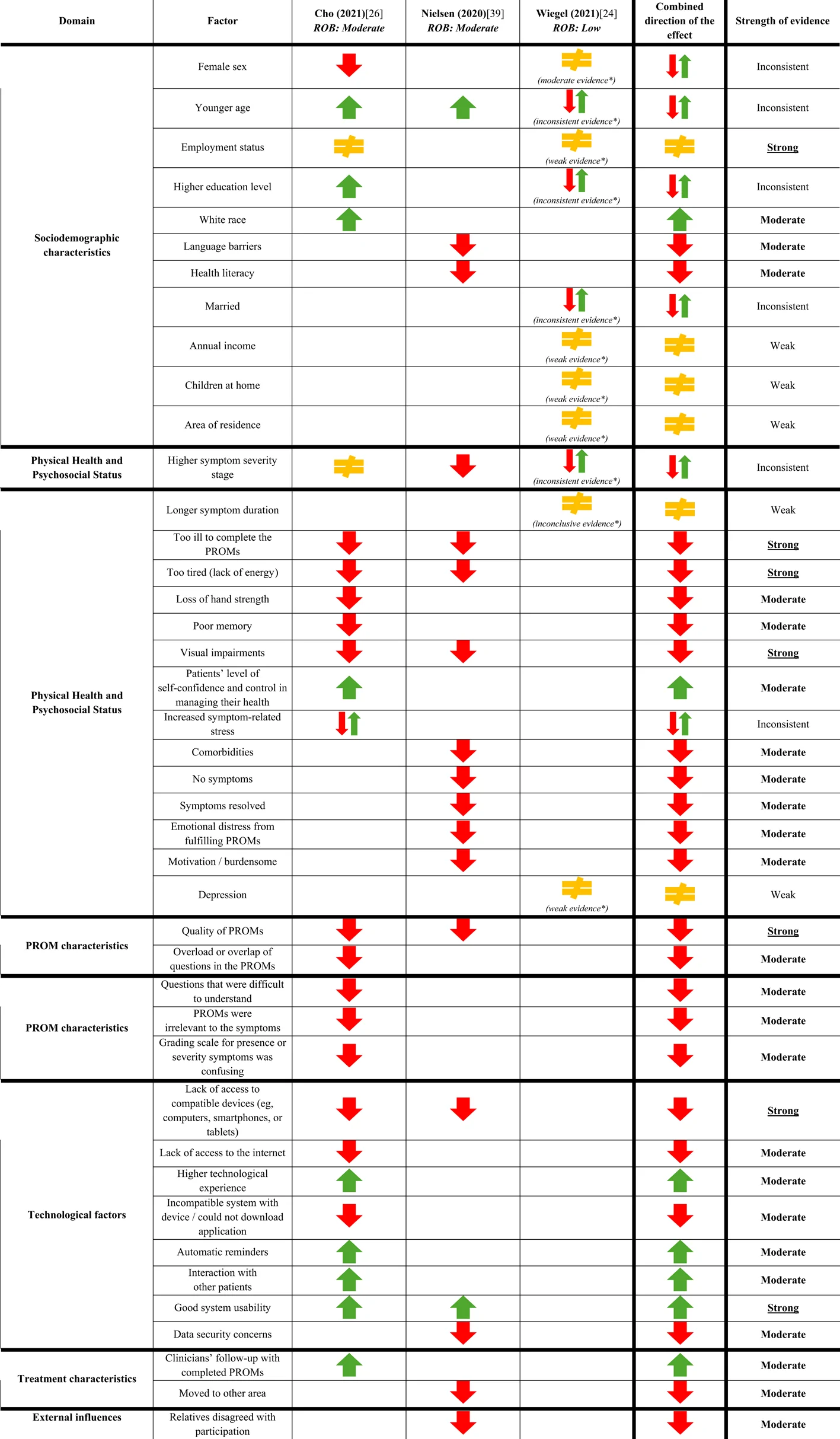
Best evidence synthesis of factors associated with (non)response to digitally collected PROMs, including, where available, the individual strength of evidence as concluded by the included reviews

PROMs = Patient-Reported Outcome Measures; ROB = Risk of Bias;  = Positive effect on response;  = Negative effect on response;  = No effect on response;  = Inconsistent effect on response: * = Author’s own defined level of evidence

### Meta-analysis

We identified 58 unique factors and their corresponding odds ratios that were potentially associated with response (see Appendix 5) [41–51]. Multiple studies reported the factors of age, BMI (body mass index), education level, employment status, race, and sex, and were thus included in the meta-analysis [41–48].

Funnel plots were used to assess publication bias (Appendix 6). The plots for BMI and employment status suggest minimal publication bias, whereas those for age, education level, race, and sex show some asymmetry, potentially indicating the presence of publication bias.

**Fig. 2:**
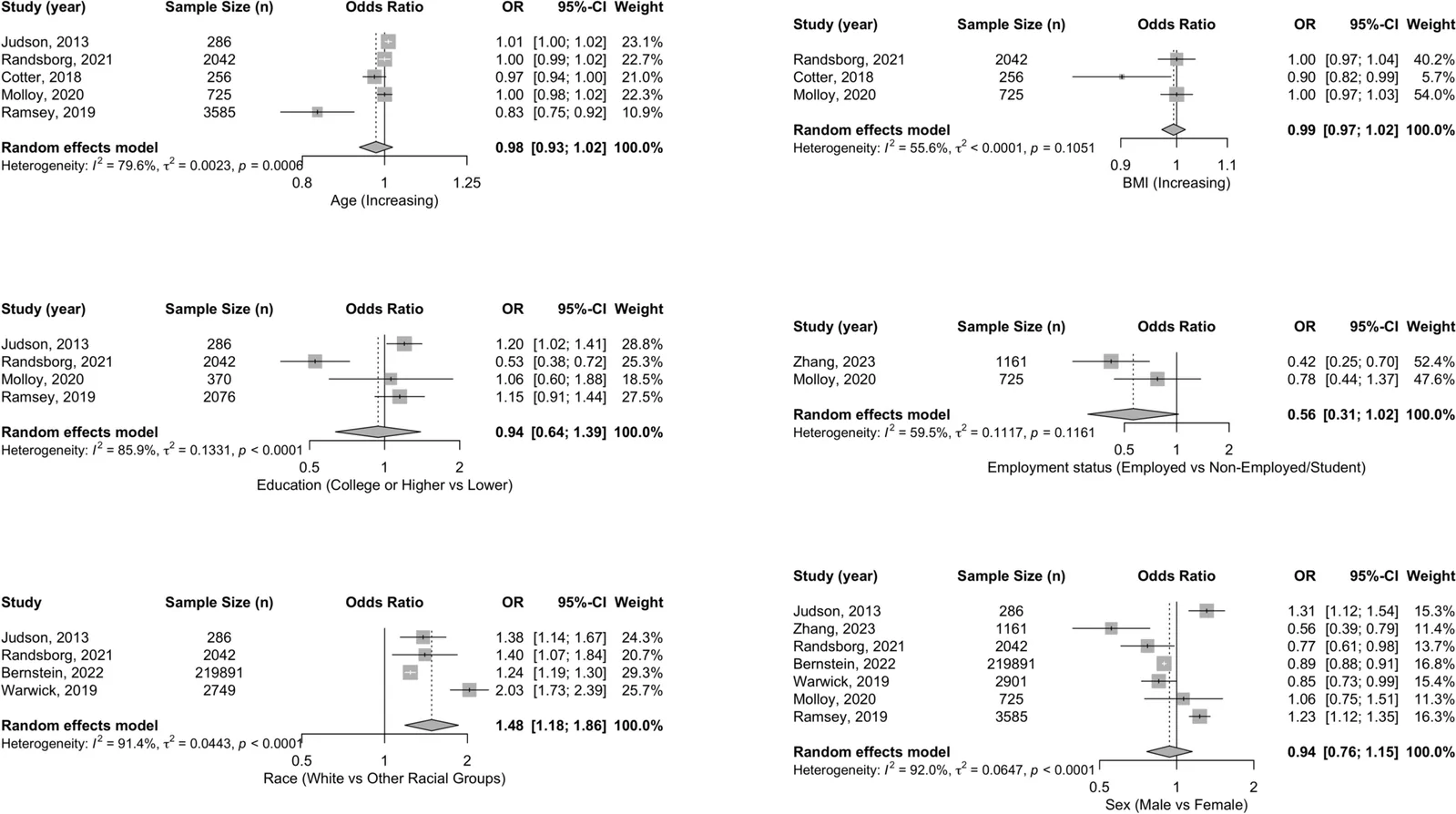
Random-effects meta-analysis: Forest plots of included factors, reporting odds ratios (OR) with 95% confidence intervals (CI). Statistical significance set at *p* < 0.05

Given that the lowest level of heterogeneity (I^2^) among the included factors was 52% (Fig. 2), we utilized a random-effects model for all variables to account for between-study variability. The random-effects models showed that age, BMI, education level, employment status, and sex were not associated with response. In contrast, patients identified as white had significantly higher response rates. The forest plots of the random-effects model are presented in Fig. 2.

### Triangulated findings

In both the best-evidence synthesis and the meta-analysis, we identified age, education, employment status, race, and sex as factors that were either associated or not associated with response (Table 4). The findings on race and employment status were consistent between the best-evidence synthesis and meta-analysis: patients identified as white had higher responses, whereas employment status did not influence their responses. Secondly, the best-evidence synthesis indicated inconsistent findings for age, education level, and sex. For education level, the meta-analysis showed no clear association (OR 0.94, 95% CI 0.64–1.39), with a wide confidence interval indicating substantial uncertainty, which is reflected in a weak level of evidence following triangulation. In contrast, the meta-analyses for age (OR 0.98, 95% CI 0.93–1.02) and sex (OR 0.94, 95% CI 0.76–1.15) showed no association, with relatively narrower confidence intervals, providing strong evidence of no association following triangulation. The triangulated findings are presented in Table 4.

**Table 4:**
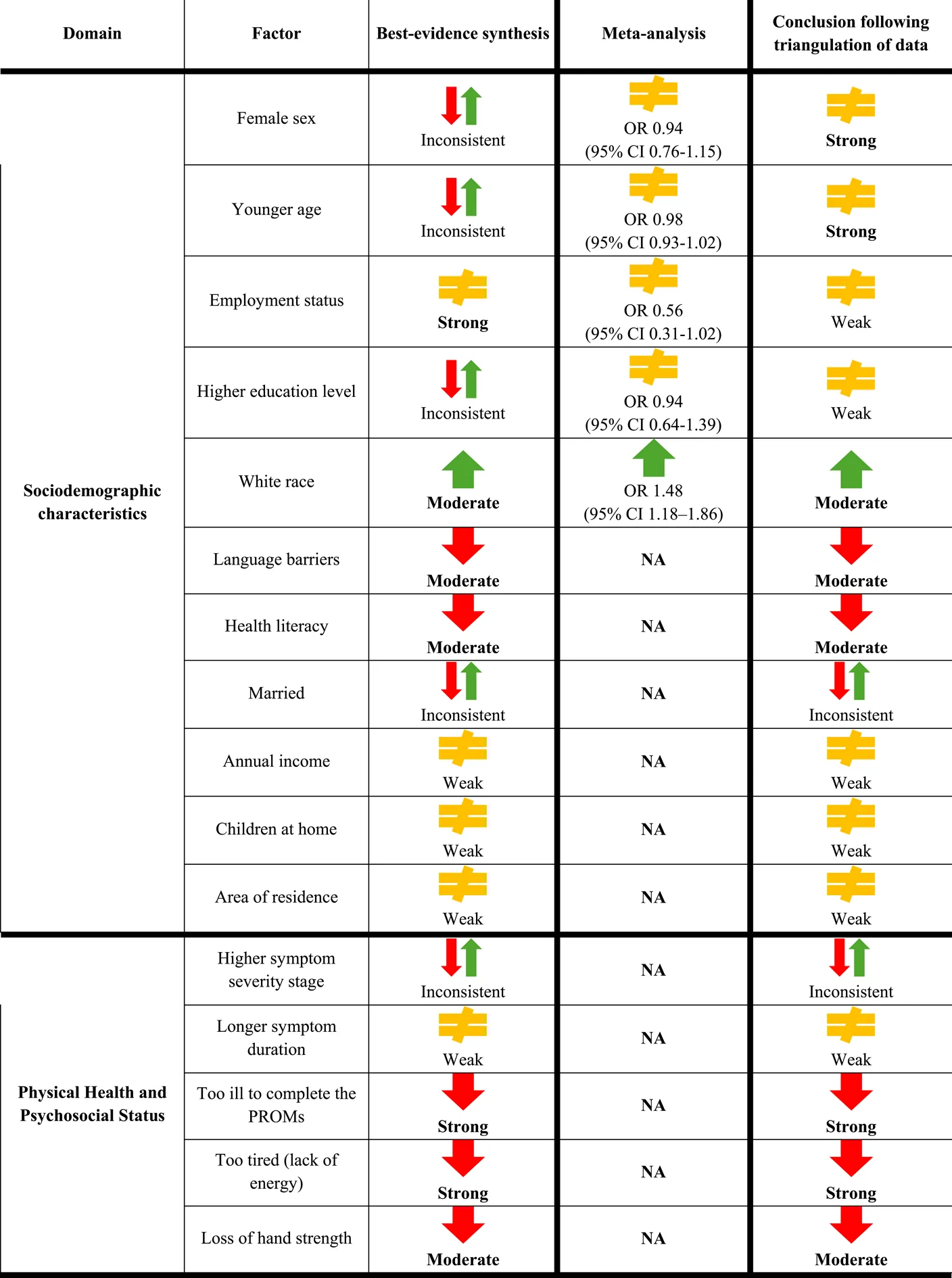

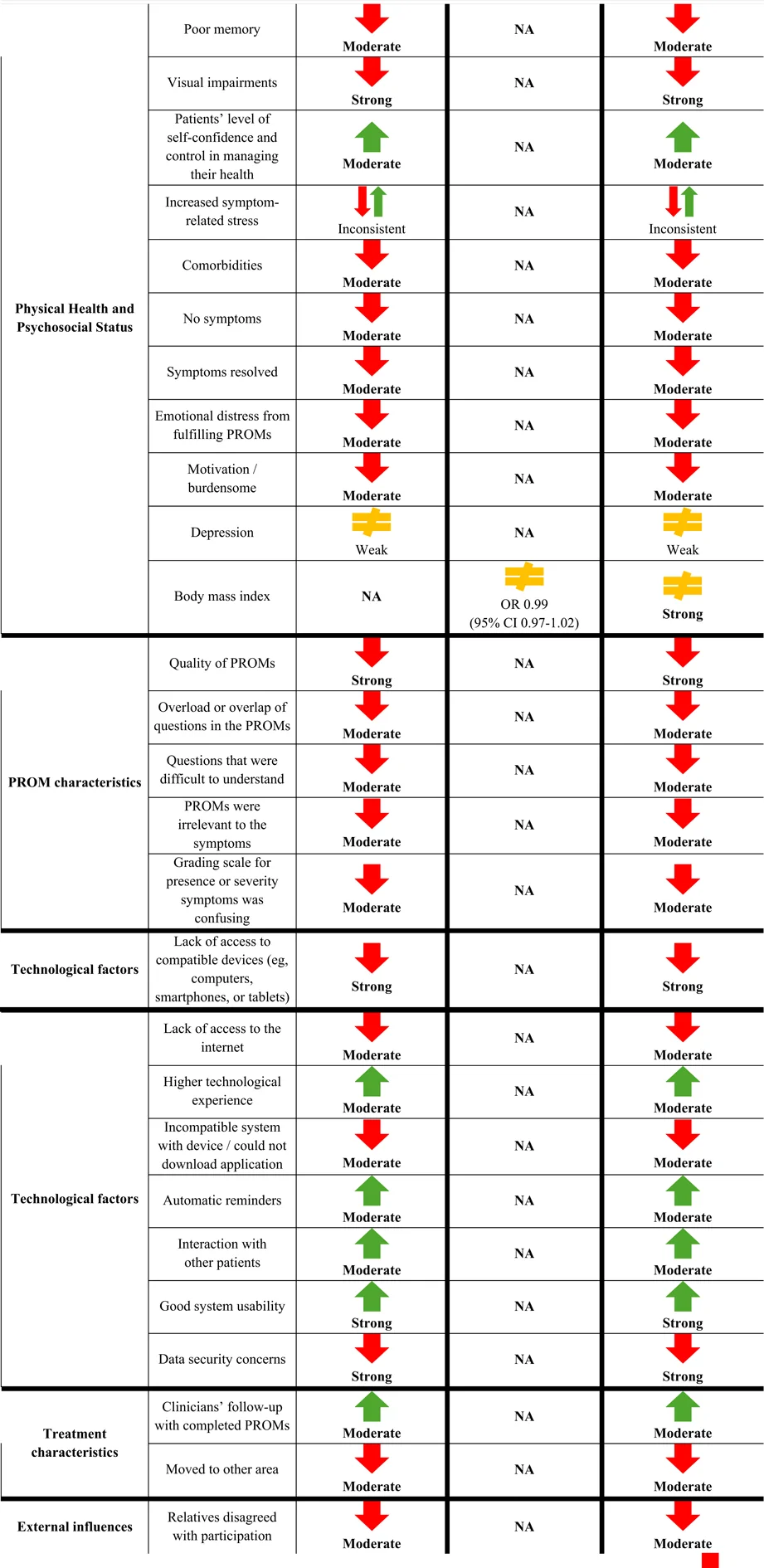
Triangulation of factors identified in the best-evidence synthesis (N = 42) and meta-analysis (N = 6)

PROMs = Patient-Reported Outcome Measures; ROB = Risk of Bias;  = Positive effect on response for the factor;  = Negative effect on response;  = No effect on response;  = Inconsistent effect on response

## Discussion

In this systematic review of systematic and scoping reviews, we identified 43 factors that were positively, negatively, or not associated with the response to digitally collected PROMs. We clustered these factors into six domains: (1) sociodemographic characteristics, (2) physical health and psychosocial status, (3) PROM characteristics, (4) technological factors, (5) treatment characteristics, and (6) external influences. Some of the factors influencing response are directly modifiable, such as the overload or overlap of questions, PROMs that were irrelevant to the symptoms, questions or grading scales that were confusing, good system usability, and clinicians’ use of PROMs in daily clinic. In contrast, other factors influencing response, such as language barriers, visual impairments, or comorbidities, cannot be modified.

More specifically, domains such as sociodemographic characteristics, physical health, and psychosocial status generally reflect factors that are not modifiable. However, it is important to emphasize that, although such factors may not be modifiable, they remain actionable. For example, language barriers can be addressed by simplifying the language to a reading level similar to that of an eleven- to twelve-year-old [23, 52]. In other words, acknowledging these factors allows tailored information provision—whether digital or in-person—to meet patients’ needs and support equal access to care, which remains a fundamental responsibility of the healthcare system [53].

There is a substantial body of existing literature that demonstrates the limited modifiability and actionability of factors associated with PROM response. Future research should therefore adopt a fundamentally different approach from the existing literature and focus exclusively on factors that can be modified or are actionable. This is needed to develop targeted interventions for improving PROM response. In other words, our findings highlight the need for a shift towards tailored interventions that target specific patient groups to improve PROM response [54]. Although several strategies to improve response were evaluated, evidence on which interventions are effective—particularly in promoting equitable response rates across diverse patient populations—remains limited [55–57]. Future studies should therefore focus on evaluating interventions to improve PROM response rates [56]. In this context, A/B testing offers a pragmatic approach to assess effectiveness in real-world settings, enabling rapid comparison of multiple strategies without overlap or contamination [58]. In such a design, participants could be randomly assigned to different versions of an application (e.g., two user interface formats or two PROMs). This enables rapid, unbiased comparison of multiple strategies without overlap or contamination, in a manner conceptually similar to randomized clinical trials. Our findings may serve as a structured overview for developing these targeted interventions and strategies.

Examples of interventions that improve PROM response could include: (1) Facilitating clinicians in following up on completed PROMs during consultations through an integrated PROM dashboard within the clinical workflow. To ensure actual use of such a dashboard, it is crucial to support its implementation with strategies such as clinician education, user-centred design, and Key Promoter Indicator-based feedback [5, 8, 59–61]. (2) Improving the user interface of the applications for patients completing PROMs and enhancing equitable access. This includes, for example, audio support or the use of visuals for patients who are illiterate. Whereas visual elements such as emoticons can simplify the grading scale [8, 23, 24, 26, 39, 60, 62]. (3) Reducing the overload and overlap of questions in PROMs can be achieved by limiting both the number of PROMs and the number of items within them. This requires a critical appraisal of the administered PROMs and the application of techniques such as computer-adaptive testing or decision tree modeling to reduce their length [8, 22, 23, 26, 61, 63]. Or (4) increasing motivation to complete PROMs by clarifying their purpose, for example, through an explanatory video, in addition to providing clear written explanations with the invitation sent [5, 8, 23, 56, 64].

### Limitations

This study has several limitations that should be considered when interpreting the results. First, most included reviews had a moderate risk of bias and lacked a best-evidence synthesis, limiting the ability to appropriately weight and interpret the strength of the evidence. This also reflects the inclusion of reviews with varying methodological approaches, including scoping reviews. Although we addressed this in our best-evidence synthesis, the levels of evidence we assigned may be over- or underestimated. Secondly, only a few studies [41–51] reported all relevant data (e.g., odds ratios, confidence intervals, etc.) required for the meta-analysis. This limits the robustness of the quantitative synthesis. Additionally, these studies did not account for multicollinearity [41–48], which may have led to unstable or biased estimates [65]. Moreover, the meta-analyses showed substantial heterogeneity (I^2^ > 50% across all factors), limiting the interpretability of the pooled estimates. Although we accounted for this heterogeneity using random-effects models, the meta-analysis should be considered exploratory, and the findings should therefore be interpreted with caution, as the observed variability likely reflects differences in study populations, PROMs, and data-collection methods.

In line with this, there was substantial variation in the PROMs, measurement time points, data collection methods, diagnoses, and interventions reported across the included reviews and among the primary studies within them. Consequently, certain factors may be more relevant to specific healthcare settings than others. This limits the generalizability of our findings and highlights the need for context-specific strategies that improve response rates. Moreover, although the identified factors were grouped into broader domains to make the findings easier to understand, these domains should be seen as a practical way to organize the results rather than a formally developed taxonomy. Furthermore, there was limited overlap among primary studies across the reviews included in the best-evidence synthesis (8 of 89 studies, 9%), which may have slightly influenced the frequency with which certain factors were reported. However, given this low level of overlap, the overall risk of substantial overrepresentation is considered low.

Accordingly, our findings may serve as a structured overview for assessing PROM responses in daily care across diverse patient populations and for developing targeted interventions, as they provide a comprehensive overview of factors influencing PROM responses.

## Conclusion

Factors associated with the response to digitally collected PROMs can be clustered into sociodemographic characteristics, physical health, psychosocial status, PROM characteristics, technological factors, treatment characteristics, and external influences. These encompass both modifiable and non-modifiable factors, which, although not always directly modifiable, can still inform targeted and tailored interventions. Although identified at the individual level, many of these factors are shaped by patients’ broader social and health-related circumstances and may therefore point to underlying structural or contextual barriers (e.g., limited access to resources, lower literacy, or health-related limitations). Such barriers may ultimately contribute to unequal access to care. Our findings offer a comprehensive and generalizable synthesis of factors, serving as a structured overview for developing targeted interventions and strategies to enhance PROM response rates and promote equitable, value-based healthcare. Future research should primarily focus on interventions to improve response rates to digitally collected PROMs across diverse patient populations.

## Supplementary Information

Below is the link to the electronic supplementary material.

Supplementary file 1 (PDF 79 KB)

Supplementary file 2 (PDF 151 KB)

Supplementary file 3 (PDF 120 KB)

Supplementary file 4 (PDF 99 KB)

Supplementary file 5 (XLSX 29 KB)

Supplementary file 6 (PDF 168 KB)

## Data Availability

The literature search strategy and the data frame used to perform the meta-analysis are available in the Appendix.
