# Supplementary material for: Factors associated with response to patient-reported outcome measures: a systematic review of systematic and scoping reviews, and meta-analyses
Source: Qual Life Res. 2026 Jun 22;35(8):213. doi: 10.1007/s11136-026-04314-9 (PMC13287233; doi:10.1007/s11136-026-04314-9)
Supplement: Supplementary file 1 — (PDF 79 KB) [file 11136_2026_4314_MOESM1_ESM.pdf]

Appendix 1. Literature search Strategy.

**Database searched**	**Platform**	**Years of coverage**	**Records**
Medline ALL	Ovid	1946 - Present	659
Embase	Embase.com	1971 - Present	1255
Web of Science Core Collection*	Web of Knowledge	1975 - Present	601
Cochrane Central Register of Controlled Trials	Wiley	1992 - Present	73
Epistemonikos	www.epistemonikos.org	1950-present	226
Additional Search Engines: Google Scholar** (200 top-ranked)			200
**Total**			**3014**

Embase 1255

('patient-reported outcome'/de OR 'patient outcomes'/de OR 'survey fatigue'/de OR 'self report'/de OR 'patient reported outcome measure'/de OR 'patient reported outcomes measurement information system'/de OR 'patient reported outcome measurement information system'/de OR (((patient* OR self*) NEAR/6 (report*) NEAR/6 (outcome*)) OR PROM OR PROMs OR ePRO OR ePROs OR (patient* NEXT/2 outcome* NEXT/2 assess*) OR ((survey* OR questionnair*) NEXT/1 (fatig* OR respons*)) OR self-report* OR self-administ*-question* OR patient-reported):ab,ti,kw) AND ('patient compliance'/de OR 'overall response rate'/de OR 'patient dropout'/de OR 'refusal to participate'/de OR completion/de OR 'completion rate'/de OR 'survey fatigue'/de OR (response* OR complian* OR adher* OR nonrespons* OR nonadher* OR noncomplian* OR refuse* OR drop-out OR dropout* OR non-use OR nonuse OR ((complet*) NEAR/3 (patient* OR report* OR PROM OR PROMs

OR survey* OR questionnair* OR ePRO OR ePROs)) OR ((survey* OR questionnair*) NEXT/1 fatig*)):ab,ti,kw NOT (medicat*-complian* OR medicat*-adheren* OR treatment-complian* OR treatment-adheren* OR therapy-complian* OR therapy-adhere*):ti) AND (telehealth/exp OR 'mobile application'/exp OR 'self-care software'/de OR 'e-mail'/de OR Internet/exp OR 'mobile phone'/exp OR telecommunication/de OR (telehealth* OR tele-health* OR mobile-health* OR mhealth* OR m-health* OR ehealth OR e-health* OR telemonitor* OR tele-monitor* OR digital* OR mobile OR web OR app OR apps OR software* OR e-mail* OR electron*-mail* OR internet* OR smartphone* OR phone OR telecommunicat* OR ICT):ab,ti,kw) AND ('systematic review'/de OR 'meta analysis '/exp OR review/exp OR (systematic-review* OR meta-analys* OR review*):ti,kw) AND [ENGLISH]/lim

Medline 659

(Patient Reported Outcome Measures/ OR exp Patient Outcome Assessment/ OR Self Report/ OR (((patient* OR self*) ADJ6 (report*) ADJ6 (outcome*)) OR PROM OR PROMs OR ePRO OR ePROs OR (patient* ADJ2 outcome* ADJ2 assess*) OR ((survey* OR questionnair*) ADJ (fatig* OR respons*)) OR self-report* OR self-administ*-question* OR patient-reported).ab,ti,kf.) AND (Patient Compliance/ OR Patient Dropouts/ OR Refusal to Participate/ OR (response* OR complian* OR adher* OR nonrespons* OR nonadher* OR noncomplian* OR refuse* OR drop-out OR dropout* OR non-use OR nonuse OR ((complet*) ADJ3 (patient* OR report* OR PROM OR PROMs OR survey* OR questionnair* OR ePRO OR ePROs)) OR ((survey* OR questionnair*) ADJ fatig*)).ab,ti,kf. NOT (medicat*-complian* OR medicat*-adheren* OR treatment-complian* OR treatment-adheren* OR therapy-complian* OR therapy-adhere*).ti.) AND (Telemedicine/ OR Mobile Applications/ OR Software/ OR Web Browser/ OR Electronic Mail/ OR exp Internet/ OR exp Cell Phone/ OR Telecommunications/ OR Digital Health/ OR (telehealth* OR tele-health* OR mobile-

health* OR mhealth* OR m-health* OR ehealth OR e-health* OR telemonitor* OR tele-monitor* OR digital* OR mobile OR web OR app OR apps OR software* OR e-mail* OR electron*-mail* OR internet* OR smartphone* OR phone OR telecommunicat* OR ICT).ab,ti,kw.) AND (Systematic Review/ OR Meta-Analysis/ OR Review/ OR (systematic-review* OR meta-analys* OR review*).ti,kf.) AND english.la.

Cochrane 73

((((patient* OR self*) NEAR/6 (report*) NEAR/6 (outcome*)) OR PROM OR PROMs OR ePRO OR ePROs OR (patient* NEXT/2 outcome* NEXT/2 assess*) OR ((survey* OR questionnair*) NEXT/1 (fatig* OR respons*)) OR self NEXT report* OR self NEXT administ* NEXT question* OR patient NEXT reported):ab,ti,kw) AND ((response* OR complian* OR adher* OR nonrespons* OR nonadher* OR noncomplian* OR refuse* OR drop NEXT out OR dropout* OR non NEXT use OR nonuse OR ((complet*) NEAR/3 (patient* OR report* OR PROM OR PROMs OR survey* OR questionnair* OR ePRO OR ePROs)) OR ((survey* OR questionnair*) NEXT/1 fatig*)):ab,ti,kw NOT (medicat* NEXT complian* OR medicat* NEXT adheren* OR treatment NEXT complian* OR treatment NEXT adheren* OR therapy NEXT complian* OR therapy NEXT adhere*):ti) AND ((telehealth* OR tele NEXT health* OR mobile NEXT health* OR mhealth* OR m NEXT health* OR ehealth OR e NEXT health* OR telemonitor* OR tele NEXT monitor* OR digital* OR mobile OR web OR app OR apps OR software* OR e NEXT mail* OR electron* NEXT mail* OR internet* OR smartphone* OR phone OR telecommunicat* OR ICT):ab,ti,kw) AND ((systematic NEXT review* OR meta NEXT analys* OR review*):ti,kw)

Web of Science 601

TS=((((patient* OR self*) NEAR/5 (report*) NEAR/5 (outcome*)) OR PROM OR PROMs OR ePRO OR ePROs OR (patient* NEAR/2 outcome* NEAR/2 assess*) OR ((survey* OR questionnair*) NEAR/1 (fatig* OR respons*)) OR self-report* OR self-administ*-question*

OR patient-reported) AND (response* OR complian* OR adher* OR nonrespons* OR nonadher* OR noncomplian* OR refuse* OR drop-out OR dropout* OR non-use OR nonuse OR ((complet*) NEAR/2 (patient* OR report* OR PROM OR PROMs OR survey* OR questionnair* OR ePRO OR ePROs)) OR ((survey* OR questionnair*) NEAR/1 fatig*)) AND (telehealth* OR tele-health* OR mobile-health* OR mhealth* OR m-health* OR ehealth OR e-health* OR telemonitor* OR tele-monitor* OR digital* OR mobile OR web OR app OR apps OR software* OR e-mail* OR electron*-mail* OR internet* OR smartphone* OR phone OR telecommunicat* OR ICT)) AND TI=(systematic-review* OR meta-analys* OR review*) NOT TI=(medicat*-complian* OR medicat*-adheren* OR treatment-complian* OR treatment-adheren* OR therapy-complian* OR therapy-adhere*) AND LA=(English)

Epistemonikos 226

(title:((PROM OR PROMs OR ePRO OR ePROs OR patient-outcome-assess* OR survey-fatig* OR survey-respons* OR self-report* OR self-administ*-question* OR patient-reported) AND (response* OR complian* OR adher* OR nonrespons* OR nonadher* OR noncomplian* OR refuse* OR drop-out OR dropout* OR non-use OR nonuse OR survey-complet* OR questionnair-complet* OR survey-fatig*) AND (telehealth* OR tele-health* OR mobile-health* OR mhealth* OR m-health* OR ehealth OR e-health* OR telemonitor* OR tele-monitor* OR digital* OR mobile OR web OR app OR apps OR software* OR e-mail* OR electronic-mail* OR internet* OR smartphone* OR phone OR telecommunicat* OR ICT)) OR abstract:((PROM OR PROMs OR ePRO OR ePROs OR patient-outcome-assess* OR survey-fatig* OR survey-respons* OR self-report* OR self-administ*-question* OR patient-reported) AND (response* OR complian* OR adher* OR nonrespons* OR nonadher* OR noncomplian* OR refuse* OR drop-out OR dropout* OR non-use OR nonuse OR survey-complet* OR questionnair-complet* OR survey-fatig*) AND (telehealth* OR tele-health* OR mobile-health* OR mhealth* OR m-health* OR ehealth OR e-health* OR telemonitor* OR

tele-monitor* OR digital* OR mobile OR web OR app OR apps OR software* OR e-mail* OR electronic-mail* OR internet* OR smartphone* OR phone OR telecommunicat* OR ICT))) AND title:(systematic-review* OR meta-analys* OR review* NOT medication-complian* NOT medication-adheren* NOT treatment-complian* NOT treatment-adheren* NOT therapy-complian* NOT therapy-adhere*)

Google Scholar

title:

review|'meta analysis' -'medication adherence' -'treatment adherence' -'therapy adherence'

keywords:

PROM|PROMs|ePRO|ePROs

response|compliance|adherence|nonresponse|nonadherence|noncompliance|refusal

mhealth|'m|e health'|ehealth|digital|mobile|web|app
