# Supplementary material for: Factors associated with response to patient-reported outcome measures: a systematic review of systematic and scoping reviews, and meta-analyses
Source: Qual Life Res. 2026 Jun 22;35(8):213. doi: 10.1007/s11136-026-04314-9 (PMC13287233; doi:10.1007/s11136-026-04314-9)
Supplement: Supplementary file 2 — (PDF 151 KB) [file 11136_2026_4314_MOESM2_ESM.pdf]

**Appendix 2.** List of excluded full-text articles with reasons

| Author (year) | Title | DOI | Reason |
|---|---|---|---|
| Daikeler 2022 | A Meta-Analysis of How Country-Level Factors Affect Web Survey Response Rates | https://dx.doi.org/10.1177/14707853211050916 | Did not include studies with digitally distributed PROMs |
| Hikmet 2003 | An investigation into low mail survey response rates of information technology users in health care organizations | https://dx.doi.org/10.1016/j.ijmedinf.2003.09.002 | Study design was not a systematic or scoping review |
| Abed 2024 | ASES and UCLA Are Responsive Patient-Reported Outcome Measures After Rotator Cuff Repair: A Systematic Review and Meta-analysis | https://dx.doi.org/10.1177/03635465231213870 | Did not assess factors associated with non-response |
| Sykes 2010 | A systematic literature review on response rates across racial and ethnic populations | https://doi.org/10.1007/bf03404376 | Did not include studies with digitally distributed PROMs |
| Anhang Price 2022 | A Systematic Review of Strategies to Enhance Response Rates and Representativeness of Patient Experience Surveys | https://dx.doi.org/10.1097/mlr.0000000000001784 | Did not include studies with digitally distributed PROMs |
| Greenup 2023 | Comparison of patient responses to telehealth satisfaction surveys in rural and urban populations in Queensland | https://dx.doi.org/10.1071/ah23116 | Study design was not a systematic or scoping review |
| Marcano Belisario 2015 | Comparison of self-administered survey questionnaire responses collected using mobile apps versus other methods | https://dx.doi.org/10.1002/14651858.MR000042.pub2 | Did not assess factors associated with non-response |
| Keith 2023 | Critical Appraisal of Electronic Surveys: An Integrated Literature Review | https://dx.doi.org/10.1891/jnm-2021-0066 | Did not assess factors associated with non-response |
| Pyper 2023 | Digital Health Technology for Real-World Clinical Outcome Measurement Using Patient-Generated Data: Systematic Scoping Review | https://dx.doi.org/10.2196/46992 | Did not include studies with digitally distributed PROMs |
| Abdelazeem 2023 | Does usage of monetary incentive impact the involvement in surveys? A systematic review and meta-analysis of 46 randomized controlled trials | https://dx.doi.org/10.1371/journal.pone.0279128 | Did not include studies with digitally distributed PROMs |
| Chan 2022 | Effect of electronic adherence monitoring on adherence and outcomes in chronic conditions: a systematic review and meta-analysis | https://doi.org/10.1371/journal.pone.0265715 | Did not assess factors associated with non-response |
| Gough 2021 | ES19.03 Understanding the Feasibility of Patient Reported Outcomes | https://dx.doi.org/10.1016/j.jtho.2021.01.043 | Conference abstract |

| Author (year) | Title | DOI | Reason |
| --- | --- | --- | --- |
| Meyer 2022 | Global Overview of Response Rates in Patient and Health Care Professional Surveys in Surgery: A Systematic Review | https://dx.doi.org/10.1097/sla.0000000000004078 | Did not include studies with digitally distributed PROMs |
| Abed 2024 | Lysholm and KOOS QoL Demonstrate High Responsiveness in Patients Undergoing Anterior Cruciate Ligament Reconstruction: A Systematic Review and Meta-analysis of Randomized Clinical Trials | https://dx.doi.org/10.1177/03635465231219966 | Did not assess factors associated with non-response |
| Garcia Abejas 2023 | Improving the Understanding and Managing of the Quality of Life of Patients With Lung Cancer With Electronic Patient-Reported Outcome Measures: Scoping Review | https://dx.doi.org/10.2196/46259 | Did not assess factors associated with non-response |
| Turner 2017 | Increasing capture of patient-reported outcomes in trauma research | https://dx.doi.org/10.1007/s11136-017-1658-6 | Conference abstract |
| Sampieri 2024 | Interventions for Concerning Patient-Reported Outcomes in Routine Cancer Care: A Systematic Review | https://dx.doi.org/10.1245/s10434-023-14576-z | Did not assess factors associated with non-response |
| Busigo Torres 2024 | Is Limited English Proficiency Associated With Differences in Care Processes and Treatment Outcomes in Patients Undergoing Orthopaedic Surgery? A Systematic Review | https://doi.org/10.1097/corr.0000000000003034 | Did not assess factors associated with non-response |
| Laupper 2023 | Late Responding in Web and Mail Surveys: A Systematic Review and Meta-Analysis | https://doi.org/10.18148/srm/2023.v17i4.8126 | Did not investigate an adult patient population |
| David 2014 | Meta-analysis of randomized controlled trials supports the use of incentives for inducing response to electronic health surveys | https://doi.org/10.1016/j.jclinepi.2014.08.001 | Did not include studies with digitally distributed PROMs |
| Bidonde 2023 | Methods, strategies, and incentives to increase response to mental health surveys among adolescents: a systematic review | https://dx.doi.org/10.1186/s12874-023-02096-z | Did not investigate an adult patient population |
| Edwards 2023 | Methods to increase response to postal and electronic questionnaires | https://dx.doi.org/10.1002/14651858.MR000008.pub5 | Did not assess factors associated with non-response |
| Van Gelder 2018 | Most response-inducing strategies do not increase participation in observational studies: a systematic review and meta-analysis | https://dx.doi.org/10.1016/j.jclinepi.2018.02.019 | Did not include studies with digitally distributed PROMs |
| Kilsdonk 2017 | Participation rates of childhood cancer survivors to self-administered questionnaires: a systematic review | https://dx.doi.org/10.1111/ecc.12462 | Did not include studies with digitally distributed PROMs |
| Lopez-Olivo 2020 | Patient adherence to screening for lung cancer in the US: a systematic review and meta-analysis | https://doi.org/10.1001/jamanetworkopen.2020.25102 | Did not assess factors associated with non-response |

| **Author (year)** | **Title** | **DOI** | **Reason** |
| --- | --- | --- | --- |
| Collado-Borrell 2022 | Patient-reported outcomes and mobile applications. A review of their impact on patients' health outcomes | https://doi.org/10.7399/fh.11830 | Did not assess factors associated with non-response |
| Dallabrida 2022 | PCR143 Meta-Analysis Shows That ePRO Compliance Averages 90% for Patients Using Their Own Smartphones in Fully Decentralized Clinical Trials | https://dx.doi.org/10.1016/j.jval.2022.04.1486 | Conference abstract |
| Wu 2022 | Response rates of online surveys in published research: A meta-analysis | https://dx.doi.org/10.1016/j.chbr.2022.100206 | Did not include studies with digitally distributed PROMs |
| Abed 2024 | Responsiveness of Patient-Reported Outcome Measures After Large Knee Articular Cartilage Transplantation: A Systematic Review and Meta-analysis | https://dx.doi.org/10.1177/03635465231196156 | Did not assess factors associated with non-response |
| Stern 2024 | Strategies to increase response rates for patient-reported outcome measures in orthopaedics: A scoping review | https://dx.doi.org/10.1002/msc.1910 | Did not include studies with digitally distributed PROMs |
| De Koning 2022 | Survey Fatigue during the COVID-19 Pandemic: An Analysis of Neurosurgery Survey Response Rates | https://dx.doi.org/10.1177/00494755221097543 | Did not investigate an adult patient population |
| Rybak 2023 | Survey mode and nonresponse bias: A meta-analysis based on the data from the international social survey programme waves 1996-2018 and the European social survey rounds 1 to 9 | https://dx.doi.org/10.1371/journal.pone.0283092 | Did not include studies with digitally distributed PROMs |
| Meyer 2022 | Survey response in colorectal surgery. A systematic review | https://doi.org/10.1016/j.sipas.2022.100068 | Did not include studies with digitally distributed PROMs |
| Van Horn 2009 | Survey Response Rates and Survey Administration in Counseling and Clinical Psychology A Meta-Analysis | https://dx.doi.org/10.1177/0013164408324462 | Did not investigate an adult patient population |
| Wilson 2024 | Survey response rates in health sciences education research: A 10-year meta-analysis | https://dx.doi.org/10.1002/ase.2345 | Did not investigate an adult patient population |
| Hubel 2025 | Sustainability and Time Trends in Electronic Patient-Reported Outcome Assessment in Routine Cancer Care: Systematic Scoping Review and Follow-Up Survey | https://doi.org/10.2196/69398 | Did not assess factors associated with non-response |
| Peasgood 2023 | Systematic Review of the Effect of a One-Day Versus Seven-Day Recall Duration on Patient Reported Outcome Measures (PROMs) | https://dx.doi.org/10.1007/s40271-022-00611-w | Did not assess factors associated with non-response |
| Skinner 2016 | The patient reported outcomes, burdens, and experiences (Probe) phase 1 study methodology and feasibility | https://doi.org/10.1186/s40814-018-0253-0 | Study design was not a systematic or scoping review |

| **Author (year)** | **Title** | **DOI** | **Reason** |
|---|---|---|---|
| Kim 2023 | The relation between eHealth literacy and health-related behaviors: systematic review and meta-analysis | https://doi.org/10.2196/40778 | Did not include studies with digitally distributed PROMs |
| He 2025 | The Reporting Completeness of Patient-Reported Outcome in Randomized Controlled Trials of Non-Small Cell Lung Cancer Could Be Improved: A Systematic Review | https://doi.org/10.1002/pon.70152 | Did not include studies with digitally distributed PROMs |
| Anderson 2024 | Understanding factors impacting patient-reported outcome measures integration in routine clinical practice: an umbrella review | https://doi.org/10.1007/s11136-024-03728-7 | Did not assess factors associated with non-response |
| Malapati 2024 | Use of patient-reported outcome measures after breast reconstruction in low- and middle-income countries: a scoping review | https://dx.doi.org/10.1186/s41687-024-00687-y | Did not assess factors associated with non-response |
| Barclay 2025 | Use of Remote Assessment Tools to Substitute Routine Outpatient Care: Scoping Review | https://doi.org/10.2196/65938 | Did not include studies with digitally distributed PROMs |
| Ekhtiari 2017 | What Makes a Successful Survey? A Systematic Review of Surveys Used in Anterior Cruciate Ligament Reconstruction | https://dx.doi.org/10.1016/j.arthro.2017.01.032 | Did not include studies with digitally distributed PROMs |
| Medway 2012 | When More Gets You Less: A Meta-Analysis of the Effect of Concurrent Web Options on Mail Survey Response Rates | https://dx.doi.org/10.1093/poq/nfs047 | Did not investigate an adult patient population |
| Benson 2023 | Why it is hard to use PROMs and PREMs in routine health and care | https://dx.doi.org/10.1136/bmjoq-2023-002516 | Study design was not a systematic or scoping review |
