# Supplementary material for: Factors associated with response to patient-reported outcome measures: a systematic review of systematic and scoping reviews, and meta-analyses
Source: Qual Life Res. 2026 Jun 22;35(8):213. doi: 10.1007/s11136-026-04314-9 (PMC13287233; doi:10.1007/s11136-026-04314-9)
Supplement: Supplementary file 3 — (PDF 120 KB) [file 11136_2026_4314_MOESM3_ESM.pdf]

Appendix 3 Risk of Bias assesment following the A MeaSurement Tool To Assess Systematic Reviews (AMSTAR).

`	**Cho et al[13] (2021)**	**Levens et al[12] (2023)**	**Nielsen et al[23] (2020)**	**Ruseckaite et al[24] (2023)**	**Van Egdom et al[25] (2019)**	**Wiegel et al[11] (2021)**
1. Was an 'a priori' design provided?	No	No	No	Yes	No	No
2. Was there duplicate study selection and data extraction?	No	Yes	No	Yes	Yes	Yes
3. Was a comprehensive literature search performed?	Yes	Yes	Yes	Yes	Yes	Yes
4. Was the status of publication (i.e. grey literature) used as an inclusion criterion?	Yes	No	Yes	Yes	Yes	Yes
5. Was a list of studies (included and excluded) provided?	Yes	Yes	Yes	Yes	Yes	Yes
6. Were the characteristics of the included studies provided?	Yes	No	Yes	No	Yes	Yes
7. Was the scientific quality of the included studies assessed and documented?	Yes	No	No	No	Yes	Yes
8. Was the scientific quality of the included studies used appropriately in formulating conclusions?	No	No	No	No	No	No
9. Were the methods used to combine the findings of studies appropriate?	Yes	Yes	Yes	Yes	Yes	Yes
10. Was the likelihood of publication bias assessed?	No	No	No	No	No	No
11. Was the conflict of interest stated?	Yes	Yes	Yes	Yes	No	Yes
**Risk of bias rating**	**Moderate**	**Moderate**	**Moderate**	**Moderate**	**Moderate**	**Low**

Reviews that achieve a score of eight or higher were considered of low risk of bias, reviews scoring between five and seven were considered of moderate risk of bias, and studies scoring less than four were considered of high risk of bias
