# Supplementary material for: Factors associated with response to patient-reported outcome measures: a systematic review of systematic and scoping reviews, and meta-analyses
Source: Qual Life Res. 2026 Jun 22;35(8):213. doi: 10.1007/s11136-026-04314-9 (PMC13287233; doi:10.1007/s11136-026-04314-9)
Supplement: Supplementary file 4 — (PDF 99 KB) [file 11136_2026_4314_MOESM4_ESM.pdf]

Appendix 4. Definitions of factors associated with response to digitally collected PROMs

Domain	Factor	Definition
**Sociodemographic characteristics**	Female sex	Defined as whether female sex was associated with higher or lower response rates.
	Younger age	Defined as whether younger age was associated with higher or lower response rates.
	Employment status	Defined as the status of being employed was associated with higher or lower response rates.
	Higher education level	Defined as whether a higher level of education was associated with higher or lower response rates.
	White race	Defined as whether individuals identifying as White were associated with higher or lower response rates.
	Language barriers	Defined as whether limited language proficiency, affecting a patient's ability to understand questionnaires or engage with the digital platform, was associated with higher or lower response rates.
	Health literacy	Defined as whether eHealth literacy, reflecting a patient's proficiency in using and interacting with digital health tools (i.e., often assessed using rating scales), was associated with higher or lower response rates.
	Married	Defined as whether being married or living with a partner was associated with higher or lower response rates.
	Annual income	Defined as whether a higher patient income is associated with higher or lower response rates.
	Children at home	Defined as whether a higher number of children living in the patient's household was associated with higher or lower response rates.
	Area of residence	Defined as whether living in an urban area was associated with higher or lower response rates.
**Physical Health and Psychosocial Status**	Higher symptom severity stage	Defined as whether more advanced disease stage (e.g., cancer stage) or greater symptom severity was associated with higher or lower response rates.
	Longer symptom duration	Defined as whether the length of time a patient has experienced symptoms was associated with higher or lower response rates.
	Too ill to complete the PROMs	Defined as whether a patient was unable to complete PROMs due to their health status, representing non-response caused by physical or clinical incapacity.
	Too tired (lack of energy)	Defined as whether fatigue or lack of energy (i.e., physical or mental exhaustion) limited a patient's ability to complete PROMs and was associated with higher or lower response rates.

Domain	Factor	Definition
Physical Health and Psychosocial Status	Loss of hand strength	Defined as whether reduced hand strength (e.g., in conditions affecting motor function such as neurological disorders), representing a physical impairment that limits a patient’s ability to operate digital devices required for PROM completion (e.g., typing or navigating interfaces), was associated with higher or lower response rates.
	Poor memory	Defined as whether impaired memory function, representing a cognitive limitation that affects a patient’s ability to remember, understand, or consistently complete PROMs (e.g., difficulties maintaining reporting routines), was associated with higher or lower response rates.
	Visual impairments	Defined as whether visual impairments (e.g., reduced eyesight), representing a physical limitation that hinders a patient’s ability to read, interpret, or interact with digital interfaces required for PROM completion, were associated with higher or lower response rates.
	Patients’ level of self-confidence and control in managing their health	Defined as whether a higher level of a patient’s perceived self-confidence and sense of control in managing their health (e.g., self-efficacy in monitoring and managing symptoms and engaging with care) was associated with higher or lower response rates.
	Increased symptom-related stress	Defined as whether higher levels of symptom-related stress (i.e., emotional burden related to experiencing symptoms) were associated with higher or lower response rates.
	Comorbidities	Defined as whether the presence of additional medical conditions alongside the primary diagnosis was associated with higher or lower response rates.
	No symptoms	Defined as whether the absence of symptoms (e.g., perceived as not sick enough) at baseline was associated with higher or lower response rates.
	Symptoms resolved	Defined as whether the resolution or reduction of symptoms over time (e.g., disease controlled) was associated with higher or lower response rates.
	Emotional distress from fulfilling PROMs	Defined as whether higher level of emotional distress caused by completing PROMs (e.g., anxiety or a burdensome reminder of illness) was associated with higher or lower response rates.
	Motivation / burdensome	Defined as whether a patient’s level of motivation to complete PROMs and the perceived burden of participation (e.g., time, effort, repetitiveness, or perceived lack of benefit) were associated with higher or lower response rates.

Domain	Factor	Definition
**Physical Health and Psychosocial Status**	Depression	Defined as whether the presence of depressive symptoms or a diagnosis of depression was associated with higher or lower response rates.
	Body mass index	Defined as whether a higher body mass index (BMI), typically calculated as weight in kilograms divided by height in meters squared, was associated with higher or lower response rates
**PROM characteristics**	Quality of PROMs	Defined as whether overall PROM design characteristics, beyond specific item-level issues (e.g., structure, personalization, and reporting frequency), were associated with higher or lower response rates.
	Overload or overlap of questions in the PROMs	Defined as whether an excessive number of questions or redundant (overlapping) items within PROMs was associated with higher or lower response rates.
	Questions that were difficult to understand	Defined as whether unclear or difficult-to-understand wording or phrasing of PROM items was associated with higher or lower response rates.
	PROMs were irrelevant to the symptoms	Defined as whether PROM items that did not reflect patients' current or relevant symptoms were associated with higher or lower response rates.
	Grading scale for presence or severity symptoms was confusing	Defined as whether unclear or confusing grading scales for symptom presence or severity were associated with higher or lower response rates.
**Technological factors**	Lack of access to compatible devices (eg, computers, smartphones, or tablets)	Defined as whether lack of access to compatible devices (e.g., computers, smartphones, or tablets) was associated with higher or lower response rates.
	Lack of access to the internet	Defined as whether lack of access to the internet required to use digital PROM systems was associated with higher or lower response rates.
	Higher technological experience	Defined as whether a higher level of prior experience or proficiency with digital technologies (e.g., computers, smartphones, or the internet) was associated with higher or lower response rates.
	Incompatible system with device / could not download application	Defined as whether incompatibility between the PROM system and a patient's device, or inability to download or install the application, was associated with higher or lower response rates.
	Automatic reminders	Defined as whether the presence of automated reminders (e.g., notifications to complete PROMs) was associated with higher or lower response rates.
	Interaction with other patients	Defined as whether the availability of interaction with other patients (e.g., peer communication within the platform) was associated with higher or lower response rates.

Domain	Factor	Definition
**Technological factors**	Good system usability	Defined as whether the perceived overall usability of the digital PROM system (e.g., ease of use, intuitive navigation, and accessibility) was associated with higher or lower response rates.
	Data security concerns	Defined as whether concerns about data privacy and security (e.g., lack of trust in digital systems or reluctance to share personal health information) were associated with higher or lower response rates.
**Treatment characteristics**	Healthcare professionals follow-up with completed PROMs	Defined as whether follow-up by healthcare professionals on completed PROMs (e.g., reviewing, acknowledging, and providing feedback on reported symptoms) was associated with higher or lower response rates.
	Moved to other area	Defined as whether relocation of the patient to another geographic area was associated with higher or lower response rates
**External influences**	Relatives disagreed with participation	Defined as whether lack of support or disagreement from relatives regarding participation in the PROM system was associated with higher or lower response rates

PROMs = Patient-Reported Outcome Measures
