# Supplementary material for: Factors associated with response to patient-reported outcome measures: a systematic review of systematic and scoping reviews, and meta-analyses
Source: Qual Life Res. 2026 Jun 22;35(8):213. doi: 10.1007/s11136-026-04314-9 (PMC13287233; doi:10.1007/s11136-026-04314-9)
Supplement: Supplementary file 6 — (PDF 168 KB) [file 11136_2026_4314_MOESM6_ESM.pdf]

Appendix 6. Funnel plots of the factors included in the meta-analysis

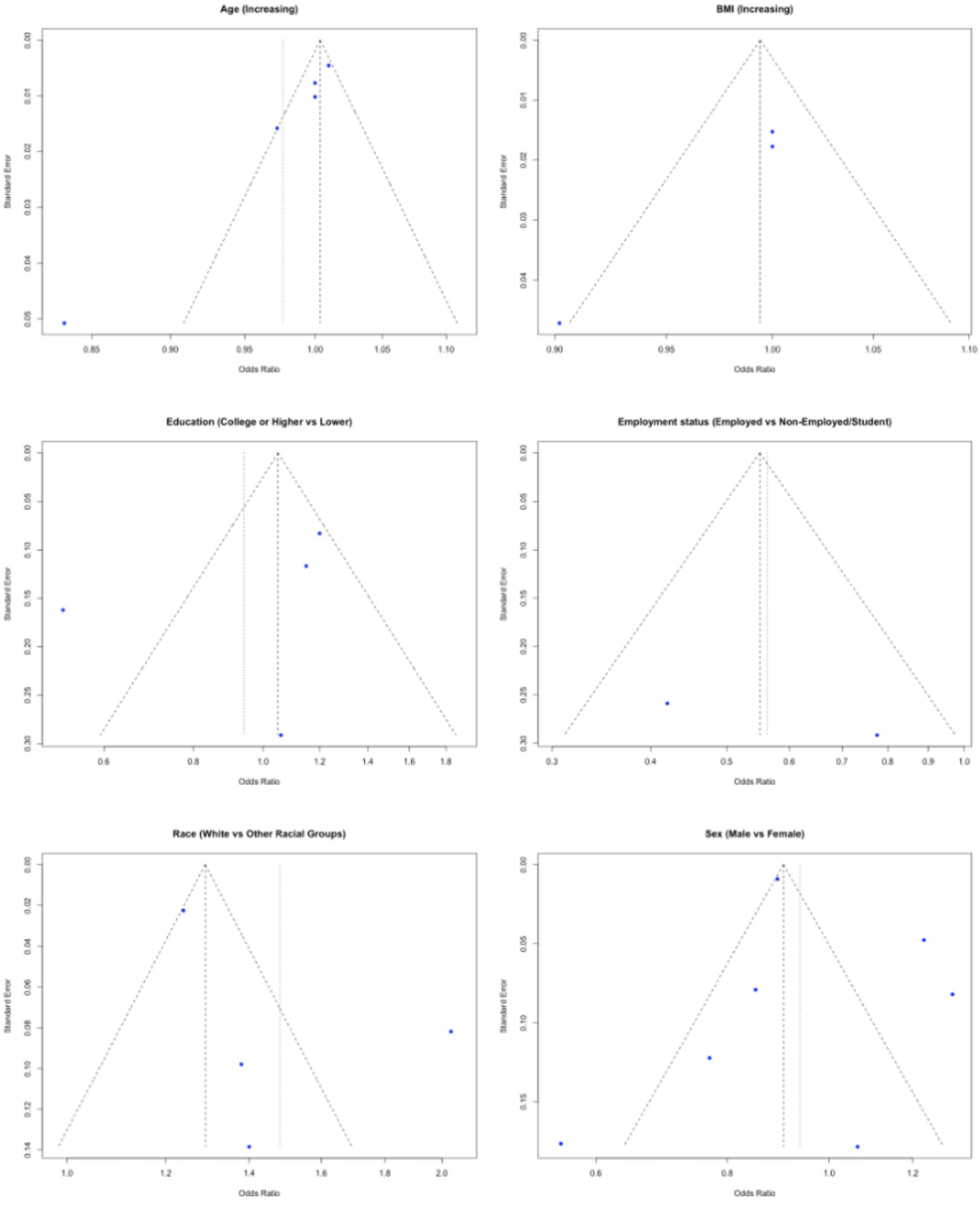

The funnel plots display the relationship between effect size (odds ratio) and study precision (standard error). Each point represents an individual study. The dotted vertical line represents the null effect (odds ratio = 1), while the dashed vertical line represents the pooled effect estimate. The diagonal lines indicate the expected 95% confidence limits around this estimate. In the absence of publication bias, studies are expected to be symmetrically distributed around the pooled effect within the funnel defined by these confidence limits. Asymmetry may indicate potential publication bias or small-study effects.
